# Bioconjugation of Serratiopeptidase with Titanium Oxide Nanoparticles: Improving Stability and Antibacterial Properties

**DOI:** 10.3390/jfb15100300

**Published:** 2024-10-07

**Authors:** Jhon Jairo Melchor-Moncada, Santiago Vasquez-Giraldo, Augusto Zuluaga-Vélez, Lina Marcela Orozco, Luz Angela Veloza, Juan Carlos Sepúlveda-Arias

**Affiliations:** 1 Grupo Infección e Inmunidad, Departamento de Ciencias Básicas, Facultad de Ciencias de la Salud, Universidad Tecnológica de Pereira, Pereira 660003, Colombia; jjmelchor@utp.edu.co (J.J.M.-M.); santivg17@utp.edu.co (S.V.-G.); azuluagav@utp.edu.co (A.Z.-V.); 2 Grupo Polifenoles, Facultad de Tecnología, Escuela de Tecnología Química, Universidad Tecnológica de Pereira, Pereira 660003, Colombia; marcely@utp.edu.co (L.M.O.); lveloza@utp.edu.co (L.A.V.)

**Keywords:** serratiopeptidase, titanium oxide, nanoparticles, antibacterial agent, bioconjugate

## Abstract

Antimicrobial resistance (AMR) poses a significant global health threat, necessitating the development of novel antibacterial strategies. Serratiopeptidase (SP), a metalloprotease produced by bacteria such as *Serratia marcescens*, has gained attention not only for its anti-inflammatory properties but also for its potential antibacterial activity. However, its protein nature makes it susceptible to pH changes and self-proteolysis, limiting its effectiveness. This study aimed to increase both the enzymatic stability and antibacterial activity of serratiopeptidase through immobilization on titanium oxide nanoparticles (TiO_2_-NPs), leveraging the biocompatibility and stability of these nanomaterials. Commercial TiO_2_-NPs were characterized using TGA/DTG, FT-IR, UV–Vis, and XRD analyses, and their biocompatibility was assessed through cytotoxicity studies. Serratiopeptidase was produced via fermentation using the C8 isolate of *Serratia marcescens* obtained from the intestine of *Bombyx mori* L., purified chromatographically, and immobilized on carboxylated nanoparticles via EDC/NHS coupling at various pH conditions. The optimal enzymatic activity was achieved by using pH 5.1 for nanoparticle activation and pH 5.5 for enzyme coupling. The resulting bioconjugate demonstrated stable proteolytic activity at 25 °C for 48 h. Immobilization was confirmed by FT-IR spectroscopy, and the Michaelis–Menten kinetics were determined. Notably, the bioconjugate exhibited two-fold greater antibacterial activity against *E. coli* than the free enzyme or TiO_2_-NPs at 1000 µg/mL. This study successfully developed a serratiopeptidase–TiO_2_ bioconjugate with enhanced enzymatic stability and antibacterial properties. The improved antibacterial activity of the immobilized enzyme presents a promising approach for developing new tools to combat antimicrobial resistance, with potential applications in healthcare, food safety, and environmental protection.

## 1. Introduction

Antimicrobial resistance (AMR) has emerged as one of the most important global health challenges of the 21st century. The World Health Organization has declared AMR one of the top ten global public health threats facing humanity [1]. By 2050, AMR is estimated to cause 10 million deaths annually, resulting in a cumulative cost of up to USD 100 trillion to the global economy [2].

The rapid emergence of multidrug-resistant bacteria, coupled with a decline in the discovery of new antibiotics, has created an urgent need for innovative antibacterial strategies [3,4,5]. This crisis has prompted researchers to investigate alternative approaches, including the examination of proteolytic enzymes [6].

Serratiopeptidase (SP), also known as serralysin or serrapeptase, is a bacterial extracellular serine protease that has garnered attention for its anti-inflammatory and potential antimicrobial properties [7,8,9]. With a molecular weight of approximately 50 kDa and containing Ca^2+^ and Zn^2+^ cofactors [10], SP is produced by various bacteria, including *Serratia marcescens*.

SP encounters various obstacles, including its low solubility, sensitivity to changes in temperature and pH, and tendency to undergo self-cleavage, all of which may have a detrimental impact on its catalytic efficiency and therapeutic potential [11,12]. To address these limitations, enzyme immobilization has emerged as a promising strategy to increase stability under various physiological and environmental conditions [13].

Covalent binding is an immobilization method that provides several advantages, such as remarkable stability, preserved properties over time, and the possibility of enzyme recovery and reuse [14]. Notably, compared with alternative methods, immobilized enzyme-based antimicrobial agents have a lower tendency to induce resistance, as revealed by previous studies [15,16].

Titanium oxide nanoparticles (TiO_2_-NPs) have gained significant interest as support structures for enzyme immobilization because of their biocompatibility, ease of fabrication, and large surface area [17]. However, TiO_2_-NPs require functionalization, such as carboxylation, to enable covalent binding with enzymes [18]. The EDC/NHS (1-ethyl-3-(3-dimethylaminopropyl) carbodiimide and N-hydroxysuccinimide) coupling strategy has proven effective for immobilizing macromolecules, forming stable amide bonds between carboxyl and amino groups [19,20].

The objective of this study was to evaluate the stability of serratiopeptidase (SP) covalently bonded to carboxyl-modified TiO_2_ nanoparticles. This approach was proposed to improve the antibacterial activity of the enzyme, which could lead to the development of new therapeutic strategies to combat antimicrobial resistance. The formation of bioconjugates is expected to pave the way for groundbreaking therapeutic solutions to address growing health issues.

## 2. Materials and Methods

### 2.1. Materials

The study materials were obtained from commercial providers. Titanium oxide nanoparticles (a mixture of rutile and anatase, nanopowder < 100 nm, product number 634,662, batch number MKCB9913), chloroacetic acid, casein from bovine milk (Bioreagent), and N-hydroxysuccinimide sodium (NHS, >97.0%) were purchased from Sigma-Aldrich (Sigma Chemical Co, Saint Louis, MO, USA). Hydroxylamine hydrochloride and 1-ethyl-3-(3-dimethylaminopropyl) carbodiimide hydrochloride (EDC) were acquired from Thermo Scientific (Thermo Fisher Scientific, Waltham, MA, USA).

### 2.2. TiO_2_-NP Characterization

The stability of the nanoparticles was investigated using thermogravimetric analysis (TGA). The analysis was performed on an SDT-650 instrument under a nitrogen atmosphere. The temperature was increased from room temperature to 800 °C at a rate of 10 °C/min. To characterize the thermal decomposition process further, the first derivative curves of the mass percentage with respect to temperature (DTG) were calculated from the TGA data.

Fourier-transform infrared spectroscopy (FT-IR) was employed to identify functional groups via a Cary 630 FTIR instrument (Agilent Technologies Inc., Santa Clara, CA, USA) equipped with a diamond-tipped attenuated total reflectance (ATR) accessory. The spectra were acquired with a resolution of 4 cm^−1^ in the range of 4000–400 cm^−1^, and 32 scans were used for background correction. Additionally, UV–Visible spectroscopy was performed using a Thermo Scientific MULTISKAN GO spectrophotometer to analyze nanoparticle dispersions (1 mg/mL) in the wavelength range of 200–800 nm, utilizing a quartz cell. This analysis allowed for the determination of surface plasmons. The optical bandgap was estimated through the application of the Tauc plot method [21] (Table 1).

The crystal structure information of the samples was obtained using a CubiX PRO (X’Pert PRO) X-ray diffraction system (Malvern Panalytical Ltd, Almelo, The Netherlands) with Cu Kα radiation (λ = 1.5406 Å). Measurements were taken in the 2θ range of 2° to 90° with a scan step time of 17.15 s and a step size of 0.02°. The samples were analyzed with the X’Pert HighScore Plus software Version 5.1, and the crystallite size was calculated. Morphology and size were analyzed by transmission electron microscopy (TEM).

### 2.3. Biocompatibility of TiO_2_-NPs

#### 2.3.1. Cell Culture

Adherent fibroblasts (HFF-1) obtained from the American Type Culture Collection (ATCC) were used. These cells were cultured in Dulbecco’s modified Eagle’s medium (DMEM) supplemented with 2.5 µg/mL amphotericin, 1% PSN (5 mg/mL penicillin, 5 mg/mL streptomycin, and 10 mg/mL neomycin), 1% 100 mM sodium pyruvate, and 10% heat-inactivated fetal bovine serum. The cells were maintained in an incubator at 37 °C and 5% CO_2_.

#### 2.3.2. Preparation of TiO_2_ Nanomaterial Suspensions

The initial suspensions of previously autoclave-sterilized nanoparticles were prepared in a supplemented DMEM containing glycerol (at a ratio of 1000 parts DMEM to 10 parts glycerol by volume) and stirred at 1500 rpm for 20 h. The concentration of the nanoparticles was prepared using serial dilutions (3000, 2500, 2000, 1500, 1000, 500, and 250 µg/mL).

#### 2.3.3. HFF-1 Cell Viability

In each well, 10,000 HFF-1 cells were added to the supplemented DMEM and incubated for 24 h under standard conditions. The nanoparticle suspensions were subsequently added for 48 h and 72 h. HFF-1 cells in DMEM supplemented with glycerol (1000:10) were used as the negative control, while 200 µL of doxorubicin at 25 µg/mL and 12.5 µg/mL served as positive controls for both 48 h and 72 h incubations. DMEM culture medium (200 µL) was used as the blank control, and titanium oxide nanoparticle suspensions without cells (200 µL) were used as sample blanks at different concentrations. After incubation, the medium was removed, and the cells were washed with 1× PBS. The cells were subsequently incubated with 200 µL of 0.5 mg/mL MTT solution for 4 h at 37 °C with 5% CO_2_. Afterward, the supernatant was removed, and 100 µL of DMSO was added to dissolve the formazan crystals formed. The absorbance was measured at a wavelength of 570 nm with a reference wavelength correction at 630 nm via a Thermo Scientific MULTISKAN GO plate spectrophotometer.

### 2.4. Production and Purification of Serratiopeptidase

A strain of *Serratia marcescens* C8 isolated from the intestine of a Colombian silkworm hybrid was utilized for serratiopeptidase production. The bacterium was inoculated at a 0.5 McFarland scale. The culture medium consisted of casein (0.5% *w*/*v*), yeast extract (0.1% *w*/*v*), soybean oil (0.3% *v*/*v*), (NH_4_)_2_HPO_4_ (0.5% *w*/*v*), KCl (0.05% *w*/*v*), NaCl (0.1% *w*/*v*), CaCl_2_·2H_2_O (0.02% *w*/*v*), MgSO_4_·7H_2_O (0.02% *w*/*v*), and ZnCl_2_ (0.01% *w*/*v*). Fermentation was carried out at 26 °C and 180 rpm for 36 h. After fermentation, the mixture was centrifuged at 4 °C and 15,500× *g* for 30 min. The supernatant was filtered through 0.45 µm and 0.22 µm membranes and concentrated via a 10 kDa ultrafiltration membrane. The bacteria-free concentrate was subsequently purified via a weak anion exchange column (Bio-Scale Mini Macro-Prep DEAE, Bio-Rad, Hercules, CA, USA). The fraction exhibiting proteolytic activity was further concentrated using ultrafiltration at 10 kDa. Purification was finalized through molecular exclusion chromatography (ENrich SEC 70, Bio-Rad, Hercules, CA, USA) at a flow of 1 mL/min [10] and monitored at 280 nm. The pure serratiopeptidase fraction was identified via molecular weight analysis using SEC and SDS-PAGE. Additionally, proteolytic activity, protein concentration, and specific activity were determined. This purified enzyme (SP) was employed in immobilization assays.

### 2.5. Proteolytic Activity and Total Protein Assay

The proteolytic activity was assessed following the method reported by Vélez et al. [10]. Azocasein served as the substrate in 25 mM Tris-HCl buffer containing 1 mM CaCl_2_ at pH 8.0. The reaction proceeded at 37 °C for 10 min, followed by inactivation with trichloroacetic acid and supernatant collection via centrifugation. An aliquot of the supernatant was mixed with sodium hydroxide, and the absorbance was measured at 440 nm. One unit of proteolytic activity (U) was defined as the amount of enzyme that catalyzed the release of one microgram of substrate per minute under standard pH and temperature conditions. The total protein concentration was determined using the Bradford commercial assay (Bio-Rad Protein Assay, Hercules, CA, USA).

### 2.6. Immobilization of the Serratiopeptidase Enzyme on Titanium Oxide Nanoparticles (TiO_2_/SP)

The carboxylation of TiO_2_-NPs followed the methodology reported by Rajaeian et al. with modifications [22]. In a stainless steel autoclave, 65 mg of TiO_2_-NPs were combined with 10 mL of deionized water. The mixture was stirred for 30 min and then sonicated for another 30 min. Subsequently, 80 mg of chloroacetic acid was added, the mixture was stirred for 5 min, and the autoclave was sealed and heated in an oven at 100 °C for 7 h. After cooling, the TiO_2_-NPs were filtered and washed with deionized water until a neutral pH was reached. Finally, the TiO_2_-NPs were lyophilized for future applications.

For the activation of the nanoparticles, 1 mg of carboxylated TiO_2_-NPs was weighed and resuspended in 250 µL of 100 mM phosphoric acid/monosodium phosphate at pH 2.6, or MES (2-(N-morpholino)ethanesulfonic acid) at pH 5.1, or sodium phosphate at pH 7.6. All buffers contained 500 mM NaCl. Subsequently, 50 µL of an EDC/NHS solution at a final concentration of 2 mM/5 mM was added, and the mixture was stirred for 2 h at room temperature. The reaction was quenched with 10 µL of β-mercaptoethanol. Ultrafiltration was then performed using a 3 kDa filter. The SP coupling was carried out one and two units above the enzyme’s isoelectric point. The nanoparticles were washed three times with 25 mM MES buffer containing 1 mM CaCl_2_ at pH 5.5 or 6.7. The TiO_2_-NPs were mixed with 0.2 mg of SP in 800 µL of 25 mM MES buffer with 1 mM CaCl_2_ and 500 mM NaCl at pH 5.5 and stirred at 4 °C for 12 h. The coupling process was repeated at pH 6.7. Finally, the reaction with hydroxylamine at a final concentration of 10 mM was carried out. Subsequently, ultrafiltration was performed using a 3 kDa filter. Afterward, the nanoparticles were washed three times with 25 mM Tris-HCl buffer containing 1 mM CaCl_2_ at pH 8.0 and stored in 300 µL of buffer. This process yielded the bioconjugate (TiO_2_/SP).

### 2.7. Characterization of the Bioconjugate TiO_2_/SP

The secondary structure of both free and immobilized serratiopeptidase enzyme was analyzed via FT-IR with an ATR cell, covering the range from 4000 to 400 cm^−1^, with 16 scans per sample. The amide I band (1700–1600 cm^−1^) was isolated for structural analysis. Baseline correction and band deconvolution of the amide I bands were subsequently performed for both enzyme forms [23,24].

The average particle size and zeta potential were measured using dynamic light scattering (DLS) (Malvern Instruments Ltd., Model ZSU3100). The samples were dispersed in distilled water at a concentration of 0.5% *w*/*v*. A 1 mL sample solution was placed in a cuvette and equilibrated at room temperature for 1 min. Measurements were taken for each sample group three times, with 16 scanning cycles per measurement.

The thermal stability of the bioconjugate (TiO_2_/SP) was assessed at 4 °C, 25 °C, and 37 °C for 48 h at pH 7.4. Proteolytic activity was measured every 6 h and expressed as a percentage of the relative activity.

Michaelis-Menten parameters were determined by measuring the proteolytic activity of SP and TiO_2_/SP using azocasein at different concentrations (0.04 mM to 2.11 mM). The enzymatic activity was evaluated under optimal conditions of pH 6 and 50 °C as previously determined by Vélez et al. [10]. The K_m_ and V_max_ values were obtained from Lineweaver-Burk plots.

### 2.8. Antibacterial Activity of the Bioconjugate TiO_2_/SP

*Escherichia coli* strain NCTC 9001(ATCC, Manassas, VI, USA) was used to evaluate the antibacterial capacity of covalently immobilized serratiopeptidase on titanium oxide nanoparticles (TiO_2_/SP), titanium oxide nanoparticles (TiO_2_), and free serratiopeptidase (SP). The experiment included kanamycin (50 µg/mL) as a positive control and untreated *E. coli* as a negative control. The samples were incubated at 37 °C, and measurements were taken each hour at 600 nm for 24 h in a 96-well plate.

### 2.9. Statistical Analysis

The results are presented as the mean ± standard deviation from the tests. Each experiment was conducted in triplicate. Data analysis and graphical representation were carried out using OriginPro 9 and GraphPad Prism 7.0 software. To evaluate the significant differences in enzyme immobilization and antimicrobial activity, a one-way analysis of variance (ANOVA) was performed with Tukey’s post hoc test. Differences were considered significant when *p* < 0.05.

## 3. Results and Discussion

### 3.1. Characterization of Titanium Oxide Nanoparticles (TiO_2_-NPs)

**Figure 1:**
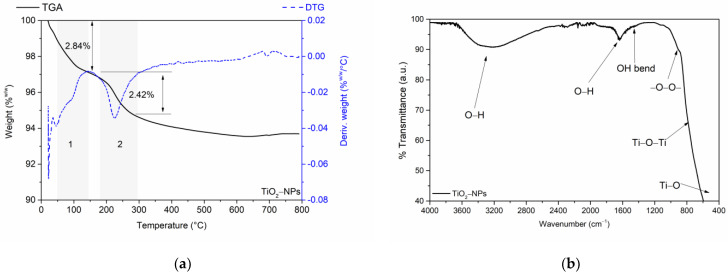
(**a**) Thermogravimetric and derivative thermogravimetric analysis of the TiO_2_ nanoparticles and (**b**) FT-IR spectrum of the TiO_2_ nanoparticles in the ATR cell.

Figure 1a shows the results of thermogravimetric analysis (TGA), and the first derivative of the TGA curve of the titanium oxide nanoparticles. This TGA curve illustrates the thermal behavior of titanium dioxide (TiO_2_) nanoparticles over a temperature range of room temperature to 800 °C (solid black line). Two distinct mass losses were observed with respect to temperature (gray shaded boxes labeled 1 and 2).

The initial steep decline of 2.84% occurs between room temperature and approximately 150 °C. This is likely due to the evaporation of physisorbed water and volatile surface-bound species. A second, more gradual weight loss of 2.42% is observed between approximately 150 and 300 °C. This may be attributed to the desorption of chemisorbed water molecules or the decomposition of surface hydroxyl groups [25]. Above 300 °C, the weight loss rate decreases significantly, indicating increased thermal stability of the TiO_2_ nanoparticles at elevated temperatures. According to some studies [26], alkaline hydrothermal synthesis in the presence of a titanium source promotes the formation of sodium titanium nanostructures. The thermogram obtained was similar to that reported by Ramanath Prabhu and colleagues when evaluating pure TiO_2_ nanopowder [27].

The derivative thermogravimetric (DTG) curve (dashed blue line in Figure 1a) corroborates these observations, showing two distinct peaks corresponding to the primary weight loss events. The first peak, centered at approximately 100 °C, is sharper and more intense, whereas the second peak, occurring between 200 °C and 300 °C, is broader and less pronounced. The total weight loss at 800 °C was approximately 6.5%, suggesting relatively high purity of the TiO_2_ nanoparticles with minimal organic contaminants or surface modifiers.

In the FT-IR spectrum of the TiO_2_-NPs (Figure 1b), a vibrational band in the 3000–3750 cm^−1^ region is observed, which is attributed to the stretching vibrations of both the O-H group bonds on the nanomaterial surface and the water present in the sample. Additionally, the presence of the -OH group can be confirmed by weak in-plane and out-of-plane bending absorptions at 1340 and 896 cm^−1^, respectively. The O-H of water was confirmed from the band at 1632 cm^−1^, corresponding to the scissoring bending vibration of water, indicating the presence of water molecules on the nanoparticle surface, either as extra or intramolecular moisture, as discussed in the TGA [28,29].

It has been reported that Ti–O and Ti–O–Ti vibrations are observed in the region from 1000 to 400 cm^−1^. Specifically, a band near 460 cm^−1^ is linked to the Ti-O bond in the anatase phase, whereas a band at 730 cm^−1^ is associated with Ti-O-Ti vibrations. Furthermore, the band between 900 and 800 cm^−1^ may be related to peroxo groups. This feature can account for the second mass loss observed in TGA, along with intramolecular water. Therefore, the presence of hydroxyl groups and metal-oxygen interactions suggest that the material corresponds to titanium oxide [30,31].

**Figure 2:**
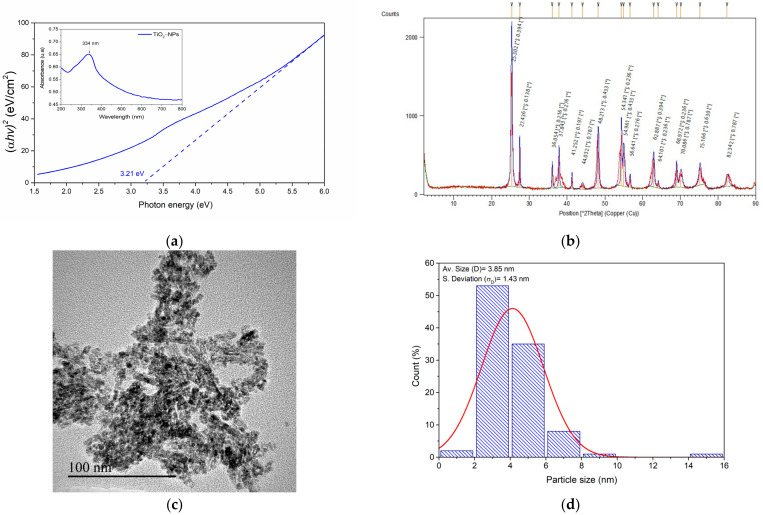
(**a**) Absorption spectrum in the UV–Vis range (inside the figure) and Tauc plot (outside the figure); (**b**) X-ray diffraction (XRD) pattern; (**c**) TEM (transmission electron microscopy) analysis; and (**d**) particle size distribution by TEM of titanium oxide nanoparticles.

On the other hand, UV–Vis spectroscopic analysis and Tauc plot treatment provide valuable insights into the optical properties of titanium dioxide nanoparticles. The UV–Vis absorption spectrum (inset of Figure 2a) reveals a distinct absorption peak at 334 nm, which can be attributed to the surface plasmon resonance (SPR) of the TiO_2_ nanoparticles. This finding aligns with several studies in the literature, which suggest that the UV absorption edge of titanium oxide nanoparticles is influenced by particle size [31,32,33,34]. Jiménez et al. demonstrated that titanium oxide nanoparticles (with an anatase structure) of 19 nm exhibited an absorption band at a wavelength of 357 nm, whereas microparticles of 1.60 µm had a band at 391 nm [33]. However, this SPR wavelength is redshifted compared with the previously reported value of 315 nm for titanium oxide [30]. This shift could be due to the presence of rutile, which generally has a lower bandgap or a higher absorption band than anatase does, as well as possible interactions between the two phases [35,36].

The main graph in Figure 2a shows the Tauc plot, which was derived from the UV–Vis data to determine the optical bandgap of the material. The x-axis represents the photon energy (eV), whereas the y-axis represents (αhν)^2^, where α is the absorption coefficient and hν is the photon energy [21]. The linear extrapolation of the absorption edge to the x-axis yields a bandgap energy of 3.21 eV for the TiO_2_ nanoparticles. This bandgap value of 3.21 eV is consistent with previous literature reports [30,37]. Additionally, on the basis of the Brus model equation [29,31,34], a particle size value of 2.60 nm was estimated via the determined bandgap value and the average bandgap values of bulk anatase and rutile (3.20 eV and 3.0 eV, respectively) [33,35,36,38], suggesting that the nanoparticles maintain the characteristic electronic structure of TiO_2_.

Further analysis, such as X-ray diffraction (XRD), was used to correlate the observed optical properties with the crystalline structure of the TiO_2_ nanoparticles. In Figure 2b (Table S1), the XRD pattern exhibits characteristic peaks corresponding to rutile (reference code: 96-900-7433) and anatase (reference code: 96-900-9087) TiO_2_ structures. The presence of sharp, well-defined peaks indicates good crystallinity of the sample [34]. The analysis performed using the X’Pert HighScore Plus software yielded a crystallite size of 29.72 nm, suggesting nanocrystalline TiO_2_ particles (Table S2) [29]. This nanoparticulate size is important because the material has a relatively large surface area [31], which is directly related to the amount of enzyme that can be immobilized. Additionally, the value obtained by UV–Vis spectroscopy is relatively small, which could serve as a basis for determining the crystallite size. Moreover, the phase identification scores of 86 for rutile and 79 for anatase indicate high confidence in the presence of both polymorphs, with rutile being slightly more dominant. The phase contents were obtained from the following formula [29]: %W_R_ = 1/(1 + 0.8(I_A_/I_R_)) × 100 and %W_A_ = (1 − W_R_) × 100, where W_R_ and W_A_ represent the contents of rutile and anatase, respectively, and I_A_ and I_R_ denote the diffraction intensities of anatase (101) at 25.30° and rutile (110) at 27.43°, respectively. The phase content was found to be 30.70% rutile and 69.30% anatase. These XRD data, combined with the crystallite size and phase scores, suggest a well-crystallized, nanostructured TiO_2_ material with a mixture of rutile and anatase phases.

On the other hand, transmission electron microscopy (TEM) analysis of TiO_2_ particles (Figure 2c) reveals clusters of nanoparticles with irregular shapes and an average size of 3.85 nm ± 1.43 nm (Figure 2d), corresponding to the primary particle size. This observation is consistent with the product’s lot specifications, which report a particle size distribution below 100 nm. However, the particles predominantly form aggregates or agglomerates. Leynen et al. reported a size of 21 nm by TEM for a mixture of anatase and rutile; however, they also observed that the particles were agglomerated on the EM grid [39]. A similar behavior was reported by Mohd for biosynthesized zinc oxide nanoparticles [40].

### 3.2. Biocompatibility of Titanium Oxide Nanoparticles

**Figure 3:**
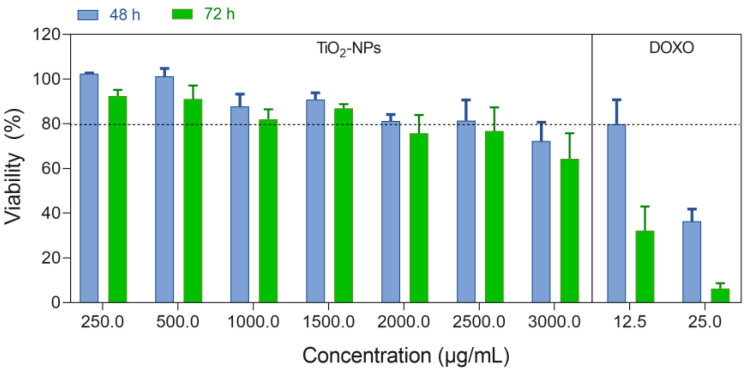
Effect of TiO_2_ nanoparticles on the viability of HFF-1 human fibroblasts after 48 h and 72 h of exposure. The dashed line indicates the level of 80% viability.

The cytotoxicity of titanium dioxide nanoparticles (TiO_2_-NPs) was evaluated in HFF-1 cells (Figure 3). The results demonstrated that TiO_2_-NPs exhibited minimal toxicity, with cell viability remaining above 80% across a concentration range of 250–2500 μg/mL at both 48 and 72 h of exposure. These findings are consistent with those of previous studies. Romoser et al. reported a low cytotoxicity of TiO_2_-NPs (25 nm) in human dermal fibroblasts [41]. Feng et al. reported a decrease in viability of approximately 18% at a TiO_2_-NP concentration of 200 µg/mL [42]. Another study reported an IC_50_ value of 168.9 µg/mL for TiO_2_-NPs in nontransformed normal human fibroblasts (GM07492). Interestingly, the same study reported no significant cytotoxic effects up to 100 µg/mL in A549 cancer cell lines after 24 h of exposure [43]. Our data indicated slightly lower cytotoxicity at comparable concentrations, which may be attributed to variations in cell type or nanoparticle characteristics. The observed cytotoxicity patterns could be related to time-dependent cellular internalization. Research has shown that NP accumulation, predominantly in the cytoplasm, can induce ultrastructural changes and rupture the cell membrane, leading to cell death. Longer exposure times typically result in increased internalization, consequently reducing cell viability or increasing cytotoxicity [44]. This finding aligns with the results obtained for the positive control, doxorubicin (DOXO), which showed significant time-dependent cytotoxicity in various cell lines [45].

Generally, as the concentration of nanoparticles increases, their cytotoxicity tends to increase due to their greater surface area, which promotes better interactions between the nanomaterial and cells. This relationship between concentration and cytotoxicity has been well documented in previous studies [46]. The biocompatibility demonstrated by these nanoparticles, as evidenced by their low cytotoxicity, suggests their potential for various biomedical applications. By using these nanomaterials to improve the properties of serratiopeptidase, we aim to develop more effective and stable therapeutic agents. This approach of using biocompatible nanoparticles to enhance enzyme properties represents a promising direction in nanomedicine, potentially leading to improved treatments and diagnostic tools.

### 3.3. Production and Purification of Serratiopeptidase (SP)

**Figure 4:**
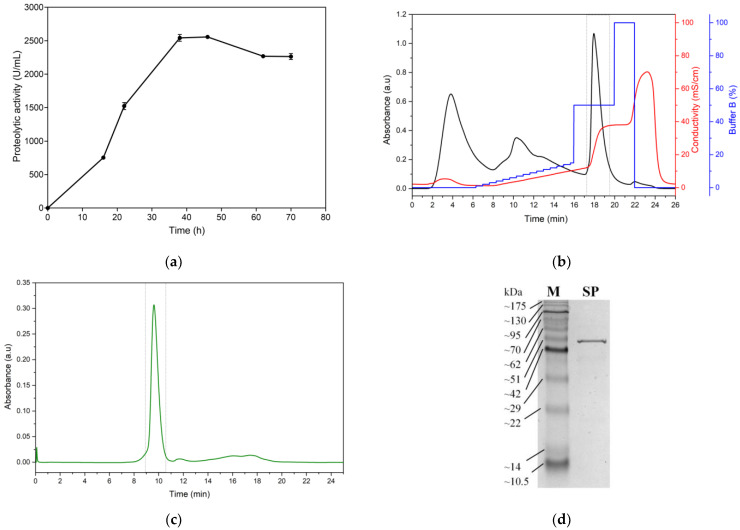
(**a**) Production kinetics of serratiopeptidase (SP) from the *Serratia marcescens* C8 isolate; enzyme purification; (**b**) chromatographic profile of weak anion exchange for the ultrafiltered supernatant from fermentation with *Serratia marcescens* (buffer B: 25 mM Tris-HCl + 1 mM CaCl_2_, pH 8.0); (**c**) size exclusion chromatography (SEC) profile of the active fraction from weak anion exchange; and (**d**) SDS-PAGE gel. M: molecular weight marker; SP: serratiopeptidase.

The production kinetics of serratiopeptidase (SP) from the *Serratia marcescens* C8 isolate demonstrated a typical growth-associated pattern of enzyme production (Figure 4a). The graph illustrates a lag phase in the first 10 h, followed by a rapid increase in proteolytic activity, reaching its peak at approximately 36 h of fermentation. The maximum SP production, quantified at 2540 U/mL proteolytic activity, was achieved under optimal conditions of 36 h fermentation time, pH 7, and a temperature of 26 °C. These parameters align well with previously reported conditions for *Serratia marcescens* fermentation, suggesting that the C8 isolate behaves similarly to other strains of this species in terms of SP production [10,47,48,49,50]. After peak production was reached at 36 h, a gradual decline in proteolytic activity was observed. This decrease could be attributed to various factors, such as nutrient depletion, accumulation of metabolic byproducts, or potential proteolytic degradation of the enzyme itself [51,52]. The shape of the production curve suggests that optimization of fermentation time is crucial for maximizing the SP yield. Extended fermentation beyond the 36 h mark may not be beneficial and could lead to reduced enzyme activity or increased production costs without corresponding increases in yield.

The chromatographic profile of weak anion exchange for the ultrafiltered supernatant revealed an efficient purification process for SP (Figure 4b). The chromatogram displays multiple peaks, indicating the separation of different protein components. The peak of interest, enclosed in the gray box, represents the proteolytically active fraction, which eluted at 17.93 min. This peak coincides with a significant increase in conductivity, ranging from 12.90 to 37.4 mS/cm, indicating the elution of SP in response to the applied salt gradient. The elution of SP at this specific point suggests that the enzyme has a net negative charge at pH 8.0, allowing its interaction with the weak anion exchange resin. The use of a buffer containing 1 M sodium chloride at pH 8.0 for elution is consistent with typical conditions for serratiopeptidase purification reported in the literature [10]. Ultrafiltration with a 10 kDa cut-off prior to chromatography is particularly relevant, as SP has a reported molecular weight of approximately 45–60 kDa. This step not only concentrated the enzyme but also effectively removed low-molecular-weight impurities and salts, thereby increasing the efficacy of the subsequent chromatographic separation [53,54]. The combination of ultrafiltration and weak anion exchange chromatography has proven to be an effective approach for SP purification. The active fraction was concentrated by ultrafiltration and evaluated using size-exclusion chromatography (SEC) at 1 mL/min (Figure 4c). The fraction obtained by SEC (gray box) was used for immobilization assays. The sharp, symmetrical shape of the SP elution peak indicates good resolution and separation from other proteins, suggesting a high degree of purity for the obtained enzyme. The purified enzyme exhibited a specific activity of 31,873.76 ± 2240.81 U/mg, a relative molecular mass of 50.35 ± 0.11 kDa determined by SDS-PAGE (Figure 4d), and 51.35 ± 0.67 kDa determined by SEC (Figure S1). These results are comparable to those reported by Koul et al., who reported a value of 51.3 kDa for serratiopeptidase produced from *S. marcescens* MES-4, with a specific activity of 13,030 U/mg [51]. Additionally, Doshi et al. reported an approximate molecular weight of 50 kDa for recombinant serratiopeptidase, with a specific activity of 8382 U/mg [55]. The purification method demonstrates good resolution and selectivity, as evidenced by the distinct peak in the chromatogram, suggesting its potential for achieving high-purity SP preparations suitable for immobilization on titanium oxide nanoparticles.

### 3.4. Immobilization of the Serratiopeptidase Enzyme on Titanium Oxide Nanoparticles (TiO_2_/SP)

**Figure 5:**
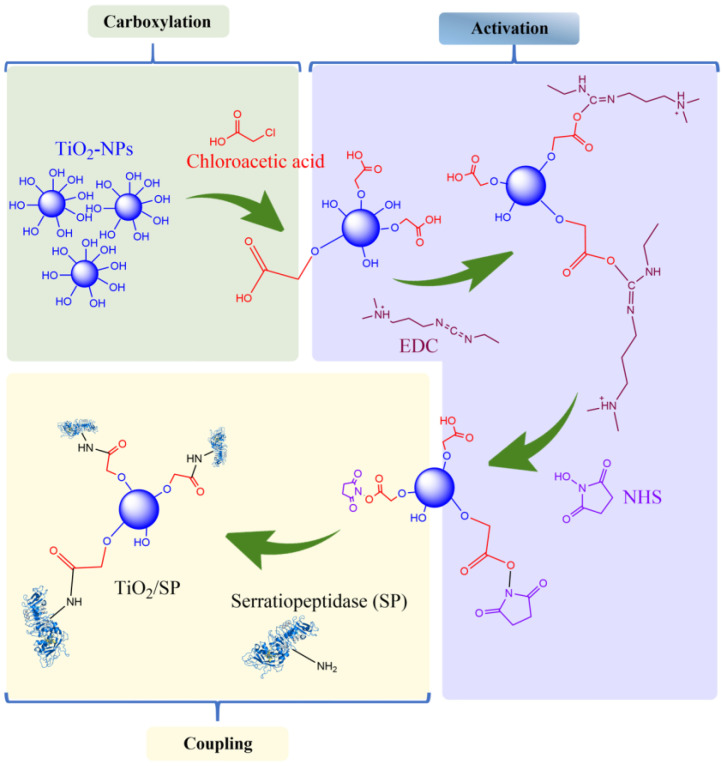
Scheme of SP immobilization reactions on carboxylated TiO_2_-NPs via the EDC/NHS strategy.

Figure 5 illustrates the step-by-step process of serratiopeptidase (SP) immobilization on titanium oxide nanoparticles via the EDC/NHS strategy. This method involves three main stages: carboxylation, activation, and coupling. The process begins with the modification of the TiO_2_-NP surface. Chloroacetic acid is used to introduce carboxyl groups onto the nanoparticle surface. The carboxylated TiO_2_-NPs are then activated via the combination of 1-ethyl-3-(3-dimethylaminopropyl)carbodiimide (EDC) and N-hydroxysuccinimide (NHS). EDC acts as a coupling agent that activates the carboxyl groups, whereas NHS creates a stable reactive intermediate. In the final step, SP is introduced to the activated nanoparticles. The amine groups of SP react with the activated carboxyl groups on the nanoparticle surface, forming stable amide bonds. This results in the covalent immobilization of SP onto the TiO_2_-NPs (TiO_2_/SP). The use of EDC/NHS chemistry for protein immobilization is well established because of its efficiency and the stability of the resulting covalent bonds [30,31].

**Figure 6:**
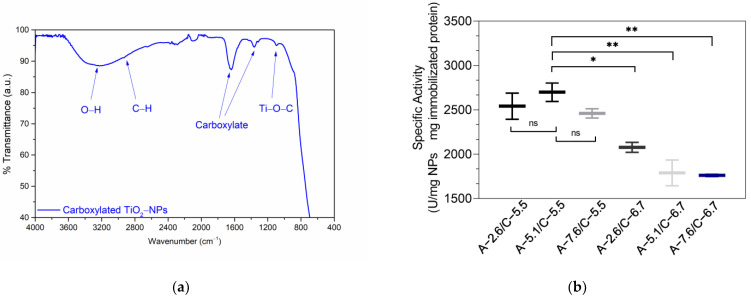
(**a**) FT-IR spectrum of carboxylated TiO_2_ nanoparticles in the ATR cell and (**b**) pH-dependent specific activity of serratiopeptidase immobilized on carboxylated TiO_2_ nanoparticles: effects of activation (A) and coupling (C) buffer pH. ns: not significant, * *p* ≤ 0.05, ** *p* ≤ 0.01.

The carboxylation of TiO_2_-NPs and the pH-dependent activity of immobilized serratiopeptidase (SP) were investigated, as shown in Figure 6. The ATR FT-IR spectrum (Figure 6a) confirmed the successful carboxylation of the TiO_2_-NPs. Pure TiO_2_-NPs exhibit a band at 3250 cm^−1^, attributed to -OH stretching vibrations of adsorbed water, corroborated by a band at 1636 cm^−1^ due to O-H bending (Figure 1b) [30].

In contrast, carboxylated nanoparticles show distinctive stretching vibrations at ~1636 cm^−1^ and 1370 cm^−1^, which are associated with C=O and C-O bonds, respectively, indicating successful carboxylation. The increase in the intensity of the hydroxyl group bands in the carboxylated material is consistent with the presence of carboxylic acids. A faint band at 2930 cm^−1^, likely due to C–H bond vibrations from chloroacetic acid residues, and a band at ~1093 cm^−1^, attributed to Ti–O–C bond bending vibrations, further confirm the carboxylation process [22,53].

The pH-dependent specific activity of the SPs immobilized on the carboxylated TiO_2_-NPs is illustrated in Figure 6b. This graph shows the effects of activation (A) and coupling (C) buffer pH on the specific activity of the enzyme. The highest specific activity (2698 ± 147.07 U/mg NP · mg immobilized protein) was achieved with an activation buffer pH of 5.1 and a coupling buffer pH of 5.5. Notably, SP immobilization was successful across various pH combinations, demonstrating the versatility of the method. The Shapiro–Wilk test revealed a normal distribution of the data, with a *p*-value of 0.4757. The one-way ANOVA analysis yielded an F-value of 16.28 and a *p*-value of 0.0020 (less than 0.05), suggesting statistically significant variations in the specific activity of immobilized serratiopeptidase across different pH groups. Tukey’s multiple comparison test results (Figure 6b) showed no significant differences within the pH 5.5 groups for coupling or within the pH 6.7 group. However, a notable difference was observed between the coupling pH of 5.5 and 6.7, particularly with A-5.1/C.5.5 (*p*-value ≤ 0.01). These findings indicate that pH is a crucial factor influencing the proteolytic activity of immobilized serratiopeptidase. The optimal pH range (5.1–5.5) aligns closely with the reported isoelectric point of SP, suggesting that this pH range facilitates effective coupling while preserving enzymatic activity [56]. Both the activation and coupling buffer pH significantly impact the resulting specific activity, highlighting the importance of pH optimization in the immobilization process. The optimal pH range (4.5–7.5) for EDC/NHS activation aligns with previous reports, validating the experimental approach [57,58,59]. These findings indicate that pH plays a crucial role in both the activation of carboxylated nanoparticles and their subsequent coupling with SP. The optimal conditions likely strike a balance between effective EDC/NHS chemistry and favorable enzyme conformation. This pH range, which is close to the isoelectric point of SP [10,60], minimizes electrostatic repulsion, potentially facilitating more efficient coupling [61]. In conclusion, this study provides valuable insights for optimizing the immobilization of SP on TiO_2_-NPs. The successful carboxylation of TiO_2_-NPs and identification of the optimal pH conditions for enzyme immobilization have contributed to the development of enhanced biocatalytic systems.

### 3.5. Characterization of TiO_2_/SP

To elucidate the decrease in proteolytic activity during immobilization, an analysis of the secondary structure of the SP enzyme was conducted via Fourier-transform infrared spectroscopy (FT-IR) with an ATR cell. Spectra were recorded from 4000 to 400 cm^−1^, with 16 scans per sample. The amide I band (1700–1600 cm^−1^) was extracted for structural analysis, followed by baseline correction and deconvolution of the constituent bands [23].

**Figure 7:**
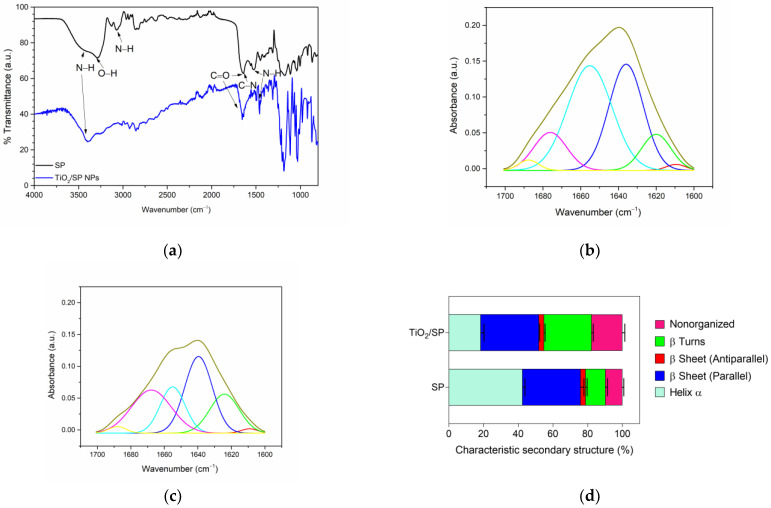

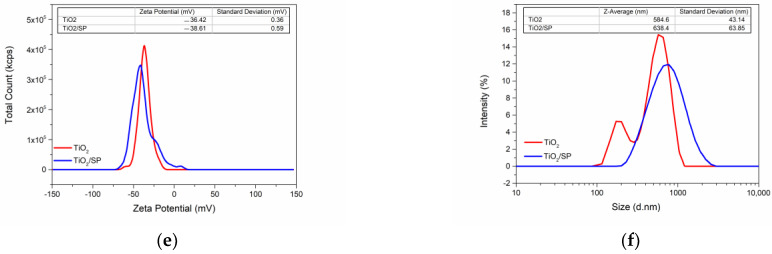
Structural analysis of serratiopeptidase and TiO_2_/SP bioconjugate: (**a**) FT-IR spectra; amide I deconvolution of (**b**) serratiopeptidase and (**c**) bioconjugate; the colors of the lines in subfigures (**b**,**c**) represent the secondary structure; (**d**) secondary structure comparison; (**e**) zeta potential; and (**f**) DLS of TiO_2_ and TiO_2_/SP bioconjugate.

Figure 7a presents the FT-IR spectra of free SP and the bioconjugate (TiO_2_/SP) recorded in the ATR cell, confirming successful SP immobilization. The spectra exhibit characteristic bands corresponding to C=O, C-N, and N-H stretching and bending vibrations, specifically amide I, amide II, and amide III, for both the nanoparticles and bioconjugate. Vibrations of the N–H and O–H bonds were observed at 3400–3050 cm^−1^, indicating the presence of immobilized SP on the nanostructured support. These findings align with those reported by Kumar et al., who observed similar bands at 3400 cm^−1^ and in the range of 1648 cm^−1^–1545 cm^−1^ when performing immobilization through amino functionalization [62]. Additionally, studies on lipases immobilized on carboxylated Fe_3_O_4_ nanoparticles have reported comparable bands in their FT-IR spectra [63,64].

Figure 7b,c illustrate the changes in the secondary structure of SP upon immobilization on titanium oxide nanoparticles activated with EDC/NHS. The deconvoluted amide I bands reveal shifts in the proportions of different secondary structural elements, suggesting conformational changes in the enzyme upon immobilization (Figure 7d). The most notable alterations included a substantial decrease in the α-helical content (from 42.45% to 18.33%) and a marked increase in the β-turn content (from 11.46% to 27.45%). Interestingly, both parallel and antiparallel β-sheet structures remained relatively unchanged, whereas nonorganized regions presented a moderate increase (from 9.75% to 17.75%). These structural modifications suggest a complex reorganization of the enzyme upon immobilization, potentially affecting its catalytic properties [65].

However, some degree of structural change is often inevitable during immobilization, and the retained activity may still be sufficient [66]. The significant loss of the α-helical structure may compromise the enzyme’s active site geometry and substrate binding capability, likely leading to decreased proteolytic activity [67]. Conversely, the increase in β-turns could contribute to a more rigid overall structure, possibly enhancing the stability against denaturation [68]. The unexpected increase in nonorganized regions hints at partial unfolding or increased flexibility in certain areas, which may have mixed effects on enzyme function and stability. Despite these changes, the preservation of β-sheet content indicates that some core structural elements remain intact, potentially allowing the enzyme to retain a degree of its native functionality [67]. These structural alterations collectively suggest a trade-off between the catalytic activity and stability of the immobilized enzyme, warranting further investigation into its kinetic parameters and long-term stability under various conditions.

The titanium dioxide nanoparticles exhibit a zeta potential of −36.42 ± 0.36 mV, as shown in Figure 7e. This value suggests that the material is moderately stable in aqueous solution [69], and the negative charge indicates that the nanoparticles are at a pH above the isoelectric point [70], meaning the surface carries a net negative charge. This property is critical for drug delivery, as the cell surface is negatively charged due to the presence of numerous anions, allowing positively charged particles to be rapidly taken up, potentially causing severe side effects and toxicity [71]. Therefore, nanoparticles with a negative surface charge could offer controlled adsorption. However, the size range obtained from dynamic light scattering measurements of the nanoparticles (Figure 7f) differs from that determined by TEM. This difference arises because TEM measures the primary particle size, whereas DLS quantifies the hydrodynamic diameter. This phenomenon has been observed in both titanium dioxide and zinc oxide nanoparticles, where the hydrodynamic size is consistently larger than the size measured by TEM [39,40,70,72,73]. As a result, in solution, the particles tend to form aggregates, leading to an increase in the hydrodynamic size of the titanium dioxide nanoparticles.

The zeta potential curve for TiO_2_/SP is slightly broader than that of TiO_2_ (Figure 7e), which may indicate greater variability in surface charge, possibly due to non-uniform functionalization and the presence of the enzyme. Additionally, since the immobilization was performed at pH 5.5, both the serratiopeptidase enzyme and the nanoparticles are above their isoelectric points [10]. Therefore, the surface charge of the bioconjugate is expected to be negative, as confirmed by the zeta potential measurement, which shifts from −36.42 mV for TiO_2_ to −38.61 mV for TiO_2_/SP. This change suggests a surface modification of the nanoparticles, consistent with enzyme immobilization. Moreover, the immobilization is further supported by the increase in the bioconjugate’s hydrodynamic size compared to TiO_2_ (Figure 7f). However, the DLS curve for the bioconjugate exhibits greater variability in size distribution, as reflected by a larger standard deviation. This increase in hydrodynamic size has also been observed during the immobilization of bovine serum albumin on ZnO via EDC/NHS coupling chemistry [72].

**Figure 8:**
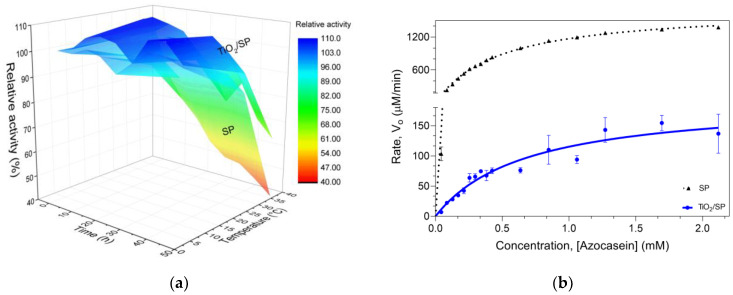
(**a**) Thermostability assessment of SP and TiO_2_/SP at various temperatures and (**b**) Michaelis-Menten kinetics of free and immobilized serratiopeptidases on titanium oxide supports.

Thermostability assessments at 37 °C (Figure 8a) demonstrated a significant increase in enzyme stability upon immobilization. After 12 h, free SP lost 38.23% ± 0.14% of its activity, whereas TiO_2_/SP maintained 101.66% ± 7.04% of its initial activity. This trend persisted over 48 h, with TiO_2_/SP losing only 36.39% of its activity compared with 60.69% for free SP. The improved thermostability aligns with the observed increase in β-turns, which likely contributes to a more rigid and stable enzyme conformation. Moreover, the loss of proteolytic activity is a natural process when the enzyme is at work temperature [48,74,75].

Michaelis-Menten kinetic analysis (Figure 8b) revealed interesting changes in enzyme behavior upon immobilization. The K_m_ value increased slightly from 0.4727 mM (free SP) to 0.6812 mM (TiO_2_/SP), suggesting a minor reduction in substrate affinity. More notably, the V_max_ decreased dramatically from 1723 µM/min (free SP) to 193.7 µM/min (TiO_2_/SP), indicating a significant reduction in the enzyme’s maximum catalytic rate when immobilized. The observed kinetic changes correlate with the structural modifications. The substantial decrease in V_max_, coupled with the slight increase in K_m_, aligns with the significant loss of α-helical content observed in the structural analysis. This conformational change likely impacts the active site geometry, reducing the catalytic efficiency [76]. The increase in β-turns and nonorganized regions may create a more compact structure that, while stabilizing the enzyme, could hinder substrate access to the active site, explaining the reduced V_max_. These findings collectively suggest a trade-off between enhanced stability and reduced catalytic activity upon immobilization of serratiopeptidase on TiO_2_ nanoparticles. Structural rigidification improves the thermal resistance but constrains the conformational flexibility necessary for optimal catalysis. Despite the lower catalytic efficiency, the significantly improved stability of TiO_2_/SP suggests potential advantages for long-term applications, such as antimicrobial activity.

**Figure 9:**
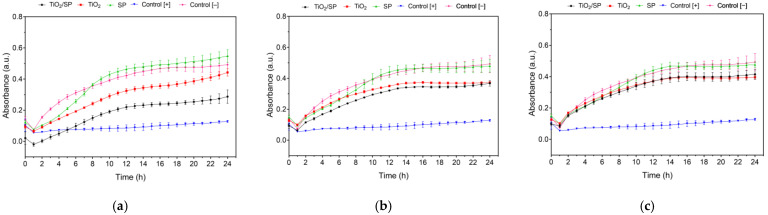
Determination of the minimum inhibitory concentration (MIC) against *E. coli* at 37 °C, measured at 600 nm, for TiO_2_/SP bioconjugates, TiO_2_ nanoparticles, serratiopeptidase (SP), kanamycin (positive control, 32 µg/mL), and no treatment (negative control) at concentrations of (**a**) 1000 µg/mL, (**b**) 500 µg/mL, and (**c**) 250 µg/mL.

*E. coli* bacteria were used to determine the minimum inhibitory concentration of the serratiopeptidase covalently immobilized on titanium oxide nanoparticles (TiO_2_/SP), titanium oxide nanoparticles (TiO_2_), and free serratiopeptidase. The incubation was carried out at 37 °C, and measurements were taken every hour at 600 nm for 24 h. As shown in Figure 9a–c, at concentrations of 1000 µg/mL, 500 µg/mL, and 250 µg/mL, the immobilized enzyme and the titanium oxide nanoparticles inhibited bacterial growth. This can be attributed to the antimicrobial properties of the titanium oxide nanoparticles [77]. To assess the behavior of the TiO_2_/SP bioconjugate at 1000 µg/mL concentration across 6, 12, and 24 h intervals, an ANOVA was performed. The findings revealed that the TiO_2_/SP bioconjugate displayed a statistically significant difference (*p* < 0.0001) when compared to the unmodified SP and TiO_2_ systems. Comparable outcomes were noted at 12 and 24 h, with the TiO_2_/SP bioconjugate showing a significant difference with TiO_2_ (*p* < 0.01). Furthermore, after 24 h, the TiO_2_/SP bioconjugate exhibited a significant difference (*p* < 0.0004) compared to the negative control, an effect not observed with free SP and TiO_2_. In particular, a greater effect was observed with the immobilized serratiopeptidase than with the nanoparticles alone. These findings demonstrate the potential of serratiopeptidase immobilized on titanium oxide nanoparticles as an antibacterial agent against *E. coli.*

Additionally, the minimum inhibitory concentration (50%) for TiO_2_/SP was 857.8 µg/mL. This was determined via the logarithm of the concentrations versus the maximum absorbance obtained at 24 h, which was normalized with respect to the controls.

The bioconjugation process alters the enzyme structure and catalytic properties but also results in significant antibacterial activity. Figure 9a–c show the minimum inhibitory concentration (MIC) for *E. coli* at 37 °C, which was measured at 600 nm over 24 h. At concentrations of 1000 µg/mL, 500 µg/mL, and 250 µg/mL, both TiO_2_/SP and TiO_2_ nanoparticles inhibited *E. coli* growth, with TiO_2_/SP exhibiting a more pronounced effect (*p*-value < 0.0001). The enhanced antibacterial efficacy of TiO_2_/SP over both free SP and TiO_2_ nanoparticles suggests a synergistic action resulting from the immobilization process. This synergy may arise from the structural changes observed in the enzyme upon immobilization, combined with the intrinsic antimicrobial properties of the TiO_2_ nanoparticles. Quantitative analysis revealed that the minimum inhibitory concentration (MIC_50_) for TiO_2_/SP against *E. coli* was 857.8 µg/mL, as determined by plotting the logarithm of concentrations against the maximum absorbance at 24 h, normalized to controls. These findings complement our earlier observations on the structural and kinetic changes in SP upon immobilization. While the catalytic efficiency (V_max_) of the enzyme decreased after immobilization, the bioconjugate exhibited enhanced thermostability and significant antibacterial activity. This multifaceted improvement in enzyme properties illustrates the potential of enzyme immobilization techniques in the development of advanced biomaterials.

Purnima et al. demonstrated that levofloxacin, when combined with serratiopeptidase, has synergistic antimicrobial and anti-biofilm activities [78]. Previous studies have shown that immobilizing serratiopeptidase on magnetite nanoparticles enhances drug permeation through membranes in vitro and significantly improves the anti-inflammatory response in carrageenan-induced paw edema in rats, even at much lower doses compared to free enzyme treatments [62]. Additionally, serratiopeptidase immobilized on cellulose nanofibers using the polyethyleneimine method demonstrated a higher inhibition of biofilm formation against *P. aeruginosa* and *S. aureus* [79]. This suggests that immobilization not only increases proteolytic activity but also enhances anti-biofilm properties. Similarly, Hanieh et al. reported that encapsulating serratiopeptidase within hyaluronic acid–lysine nanogels improves its stability under varying environmental conditions and increases enzymatic efficiency [80]. In this context, the improved antibacterial activity of the TiO_2_/SP bioconjugate observed in our study may result from the accumulation of the bioconjugate on bacterial cell surfaces, which prolongs enzyme–bacteria interactions and disrupts protein synthesis critical for bacterial invasion [80,81,82]. Furthermore, the inherent antibacterial properties of titanium oxide have been demonstrated in pathogenic *Escherichia coli* strains isolated from clinical samples, supporting the potential dual action of the TiO₂/SP bioconjugate [83]. The enhanced ability of TiO_2_/SP to inhibit bacterial growth highlights the importance of enzyme modification for the development of innovative antimicrobial therapies.

The antibacterial properties of TiO_2_/SP, combined with its improved thermal stability, open new possibilities for applications in various fields. Potential areas of application include biomedical devices, where the bioconjugate could create self-sterilizing surfaces on medical implants or instruments; water treatment systems, leveraging its ability to inhibit *E. coli* growth; food packaging [84], where its antibacterial nature could extend food shelf-life; and wound healing [85], where the combination of proteolytic activity and antibacterial properties could aid in both wound debridement and infection prevention.

## 4. Conclusions

This study demonstrated the successful enhancement of serratiopeptidase (SP) through its immobilization on biocompatible titanium oxide nanoparticles (TiO_2_-NPs), aligning with our aim to develop a more stable and effective therapeutic agent. The purification of SP from *Serratia marcescens* and its subsequent covalent immobilization on carboxylated TiO_2_-NPs via the EDC/NHS strategy resulted in a bioconjugate (TiO_2_/SP) with significantly improved properties. The TiO_2_/SP bioconjugate exhibited remarkable thermal stability, retaining 63.61% of its initial activity after 48 h at 37 °C, whereas only 39.31% of the free enzyme was stable. This enhanced stability, confirmed through FT-IR analysis and kinetic studies, suggests a longer shelf-life and improved cost-effectiveness for potential large-scale production. Notably, the immobilized enzyme demonstrated significant antibacterial activity against *Escherichia coli*, surpassing the effects of both free SP and TiO_2_-NPs alone. The synergistic effect of the antibacterial properties, coupled with the biocompatibility and low toxicity of TiO_2_-NPs, presents new opportunities for biomedical applications. Although a reduction in catalytic efficiency was observed, the gains in stability and antibacterial properties presented a favorable trade-off, highlighting the multifunctional nature of the bioconjugate. The structural changes induced by immobilization, particularly the α-helical content and β-turns, provide insights into the mechanism underlying these enhanced properties. This study underscores the potential of enzyme immobilization strategies for creating advanced biomaterials with diverse functionalities. The use of biocompatible TiO_2_-NPs as a support not only enhances the stability and bacterial efficacy of SP but also ensures its potential for safe therapeutic use.

## Figures and Tables

**Table 1:** Equations and constants for optical bandgap determination via Tauc plot analysis.

Tauc Equation ${\left(\alpha \times h\times v\right)}^{\gamma }=A\times (hv-Eg)$		Units
A	Absorbance	a.u
α	Absorption coefficient=2.302×A	${cm}^{-1}$
h	$Planck’s\;constant=4.135\times 10^{-15}$	eV.s
c	$Speed\;of\;light=3\times 10^{8}m/s$	m/s
v	$Frequency=\frac{c}{wavelength}$	$s^{-1}$
γ	For direct electronic transitions, it takes a value of 2; for indirect transitions, it is 1/2.	a.u
Eg	Band gap	eV

## Data Availability

The original contributions presented in this study are included in the article/Supplementary Materials. Further inquiries can be directed to the corresponding author.

## Supplementary Materials

The following supporting information can be downloaded at https://www.mdpi.com/article/10.3390/jfb15100300/s1, Figure S1: Calibration curve for determination of relative molecular mass by SEC. As standards, conalbumin, chicken ovalbumin, soybean trypsin inhibitor, and equine myoglobin were used; Table S1: XRD data peak list for TiO_2_; Table S2: Calculated crystallite sizes of titanium oxide nanoparticles via the Scherrer calculator in X’Pert HighScore Plus software (mean crystallite size: 29.72 nm).
