# Supplementary material for: Bioconjugation of Serratiopeptidase with Titanium Oxide Nanoparticles: Improving Stability and Antibacterial Properties
Source: J Funct Biomater. 2024 Oct 7;15(10):300. doi: 10.3390/jfb15100300 (PMC11508812; doi:10.3390/jfb15100300)
Supplement: Supplementary file 1 [file jfb-15-00300-s001.zip › jfb-3161999-supplementary.pdf]

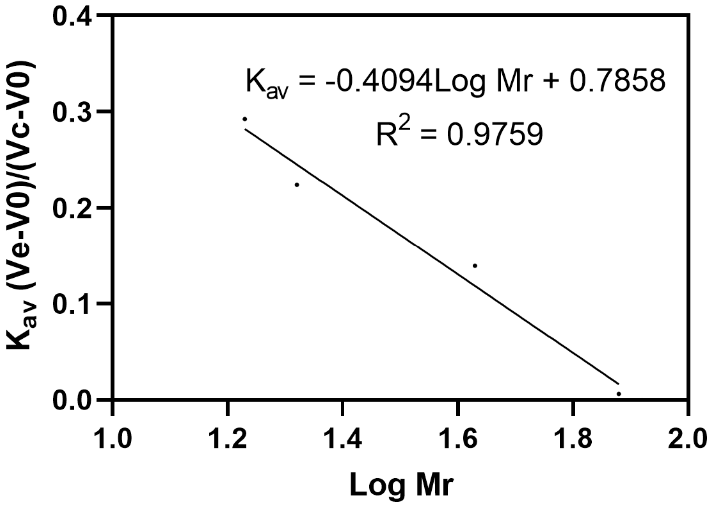

Figure S1: Calibration curve for determination of relative molecular mass by SEC. As standards, conalbumin, chicken ovalbumin, soybean trypsin inhibitor, and equine myoglobin were used

Table S1. XRD data peak list for TiO_2.

Pos. [°2Th.]	Height [cts]	FWHM Left [°2Th.]	d-spacing [Å]	Rel. Int. [%]
25.302350	1461.660000	0.393600	3.52002	100.00
27.435570	518.903600	0.137760	3.25098	35.50
36.053790	264.872500	0.216480	2.49121	18.12
37.842800	401.054400	0.275520	2.37745	27.44
41.252130	171.988700	0.196800	2.18850	11.77
44.032140	57.904720	0.787200	2.05656	3.96
48.212760	571.384800	0.432960	1.88755	39.09
54.341050	740.518800	0.236160	1.68828	50.66
54.961250	411.112500	0.432960	1.67069	28.13
56.640530	146.992400	0.275520	1.62508	10.06
62.887180	349.184700	0.393600	1.47786	23.89
64.101040	80.498170	0.236160	1.45277	5.51
68.971820	305.502700	0.236160	1.36159	20.90
70.085810	170.327000	0.787200	1.34265	11.65
75.166180	207.600000	0.629760	1.26401	14.20
82.342380	113.126800	0.787200	1.17108	7.74

Table S2. Calculated crystallite sizes of titanium oxide nanoparticles using the Scherrer calculator in X'Pert HighScore Plus software (Mean crystallite size: 29.72 nm)

B obs. (°2Th)	B std. (°2Th)	Peak pos. (°2Th)	B struct. (°2Th)	Crystallite size (nm)
0.394	0.008	25.302	0.386	21.1
0.138	0.008	27.436	0.130	62.9
0.216	0.008	36.054	0.208	40.2
0.276	0.008	37.843	0.268	31.3
0.197	0.008	41.252	0.189	44.9
0.787	0.008	44.032	0.779	11.0

0.433	0.008	48.213	0.425	20.5
0.236	0.008	54.341	0.228	39.2
0.433	0.008	54.961	0.425	21.1
0.276	0.008	56.641	0.268	33.7
0.394	0.008	62.887	0.386	24.1
0.236	0.008	64.101	0.228	41.1
0.236	0.008	68.972	0.228	42.3
0.787	0.008	70.086	0.779	12.5
0.63	0.008	75.166	0.622	16.1
0.787	0.008	82.342	0.779	13.5
